# Mutual reinforcement of inflammation and carcinogenesis by the *Helicobacter pylori* CagA oncoprotein

**DOI:** 10.1038/srep10024

**Published:** 2015-05-06

**Authors:** Nobumi Suzuki, Naoko Murata-Kamiya, Kohei Yanagiya, Wataru Suda, Masahira Hattori, Hiroaki Kanda, Atsuhiro Bingo, Yumiko Fujii, Shin Maeda, Kazuhiko Koike, Masanori Hatakeyama

**Affiliations:** 1 Division of Microbiology, Graduate School of Medicine, The University of Tokyo, Tokyo, Japan; 2 Department of Gastroenterology, Graduate School of Medicine, The University of Tokyo, Tokyo, Japan; 3 Center for Omics and Bioinformatics, Graduate School of Frontier Sciences, The University of Tokyo, Kashiwa, Japan; 4 Division of Pathology, Cancer Institute, Japanese Foundation for Cancer Research, Tokyo, Japan; 5 Gastroenterology Division, Yokohama City University Graduate School of Medicine, Yokohama, Japan

## Abstract

*Helicobacter pylori cagA*-positive strain delivers the CagA oncoprotein into gastric epithelial cells and at the same time elicits stomach inflammation. To experimentally investigate the pathophysiological interplay between CagA and inflammation, transgenic mice systemically expressing the bacterial *cagA* gene were treated with a colitis inducer, dextran sulfate sodium (DSS). Compared with control mice, DSS-induced colitis was markedly deteriorated in *cagA*-transgenic mice. In the colonic epithelia of *cagA*-transgenic mice, there was a substantial decrease in the level of IκB, which binds and sequesters NF-κB in the cytoplasm. This IκB reduction was due to CagA-mediated inhibition of PAR1, which may stimulate IκB degradation by perturbing microtubule stability. Whereas the CagA-mediated IκB reduction did not automatically activate NF-κB, it lowered the threshold of NF-κB activation by inflammogenic insults, thereby contributing to colitis exacerbation in *cagA*-transgenic mice. CagA also activates inflammasomes independently of NF-κB signaling, which further potentiates inflammation. The incidence of colonic dysplasia was elevated in DSS-treated *cagA*-transgenic mice due to a robust increase in the number of pre-cancerous flat-type dysplasias. Thus, CagA deteriorated inflammation, whereas inflammation strengthened the oncogenic potential of CagA. This work revealed that *H. pylori* CagA and inflammation reinforce each other in creating a downward spiral that instigates neoplastic transformation.

Infection with *Helicobacter pylori cagA*-positive strains plays a key role in the development of gastric carcinoma, the second-leading cause of cancer death^1^,^2^. The *cagA*-encoded CagA protein is delivered into gastric epithelial cells via bacterial type IV secretion^3^. Delivered CagA undergoes tyrosine phosphorylation by Src family kinases or c-Abl kinase at the C-terminal EPIYA motifs. Upon tyrosine phosphorylation, CagA acquires the ability to interact with and thereby deregulate the pro-oncogenic SHP2 tyrosine phosphatase, causing aberrant activation of the Ras-Erk MAP kinase signaling pathway^4^. CagA also binds to the polarity-regulating serine/threonine kinase partitioning-defective 1 (PAR1)/microtubule affinity-regulating kinase (MARK) and inhibits the kinase activity to cause junctional and polarity defects independently of tyrosine phosphorylation^5^. Oncogenic potential of CagA has been corroborated by transgenic studies in animal models including the mouse, drosophila, and zebrafish^6^,^7^,^8^. Transgenic mice systemically expressing CagA (hereafter denoted as *cagA*-Tg mice) spontaneously develop gastrointestinal or hematological malignancy by one and a half years of age^6^. Induction of neoplastic lesions in *cagA*-Tg mice requires CagA tyrosine phosphorylation, indicating a critical role of CagA-deregulated SHP2 in *in vivo* tumorigenesis. Unlike *H. pylori-*associated human gastric cancer cases^2^, however, gastrointestinal neoplasias that develop in *cagA*-Tg mice are not associated with overt inflammation^6^, suggesting that CagA can induce tumors in a cell-autonomous fashion.

Chronic inflammation provides a microenvironmental milieu that fosters cancer-predisposed cells^9^. The NF-κB transcription factor is a primary mediator of the inflammatory response, and a number of studies have demonstrated a pivotal role of NF-κB in linking inflammation and cancer^10^. In unstimulated cells, NF-κB is sequestrated in the cytoplasm upon its interaction with the NF-κB inhibitor IκB. Infectious microbes as well as proinflammatory cytokines activate IκB kinase (IKK), which in turn phosphorylates IκB and thereby promotes proteasome-dependent degradation of IκB. A decrease in the level of cellular IκB below a certain threshold triggers nuclear translocalization of NF-κB and subsequent induction of inflammation-associated genes. *H. pylori* activates NF-κB in both epithelial and immune cells via bacterial lipopolysaccharide (LPS), peptidoglycan, or type IV secretion components^11^,^12^,^13^. CagA has also been reported to activate NF-κB through multiple distinct signaling pathways in epithelial cells into which it has been delivered^14^, although the actual contribution of CagA in NF-κB activation during *H. pylori* infection remains unclear.

Given that *cagA*-positive *H. pylori* not only delivers CagA but also induces chronic inflammation in the stomach mucosa^1^,^2^, it is tempting to speculate that inflammation synergizes with the bacterial oncoprotein in the development of neoplasias. In this regard, *cagA*-Tg mice provide a unique opportunity to experimentally investigate pathophysiological interplay between CagA and inflammation, which should shed new light on the mechanism underlying CagA-mediated gastric carcinogenesis. By treating *cagA*-Tg mice with dextran sulfate sodium (DSS), a colitis inducer^15^, we found in this work that CagA and inflammation reciprocally reinforce their actions to instigate carcinogenesis.

## Results

### DSS-induced colitis in *cagA*-transgenic mice

We previously reported that a small fraction of C57BL/6J mice expressing the *H. pylori cagA* gene under the control of the *β-actin* promoter (*cagA*-Tg mice) spontaneously develop gastrointestinal and hematological neoplasias by 72 weeks of age^6^. We hypothesized that the low incidence of neoplasia in *cagA*-Tg mice was due to 1) a low level of CagA expression and/or 2) the lack of accompanying inflammation. To determine whether inflammation can potentiate CagA-mediated tumorigenesis, we sought to elicit chronic inflammation in *cagA*-Tg mice. To this end, we first infected *cagA*-Tg mice with an *H. pylori cagA*-negative strain or *Helicobacter felis*. We noticed, however, that it was technically difficult to consistently infect mice with *Helicobacter* spp. so that they chronologically develop mucosal inflammation to similar extents. We were therefore hesitant to perform a large-scale *Helicobacter* infection experiment (n > 100 mice for each experimental group). In a previous work, we found that neoplastic lesions are induced not only in the stomach but also in the intestine of *cagA*-Tg mice^6^. This observation indicated that intestinal epithelial cells are also sensitive to the oncogenic action of CagA. In *cagA*-Tg mice, intestinal expression of *cagA* mRNA was among the highest in various tissues/organs examined^6^. The CagA protein was also detectable in the colon of *cagA*-Tg mice (Figure S1). DSS is a chemical compound that has been widely utilized to induce colitis in rodents^15^,^16^. Since colonic inflammation elicited by DSS was rather uniform in magnitude among mice when compared to stomach inflammation induced by *Helicobacter* infection, we decided to investigate functional interactions between CagA and inflammation by treating *cagA*-Tg mice with DSS. In this experiment, we focused on the development of pre-neoplastic/neoplastic lesions in the mouse colon and thus employed the long-lasting DSS treatment, which maintains chronic colitis and induces colonic dysplasia in rare cases^15^. Specifically, *cagA*-Tg mice and control mice (C57BL/6J mice) at 6 weeks of age were administrated 2% DSS for 4 days, followed by 17 days of distilled water for recovery. The DSS treatment (21 days/cycle) was repeated for 14 cycles and the mice were subjected to autopsy at 48 weeks of age.

**Figure 1:**
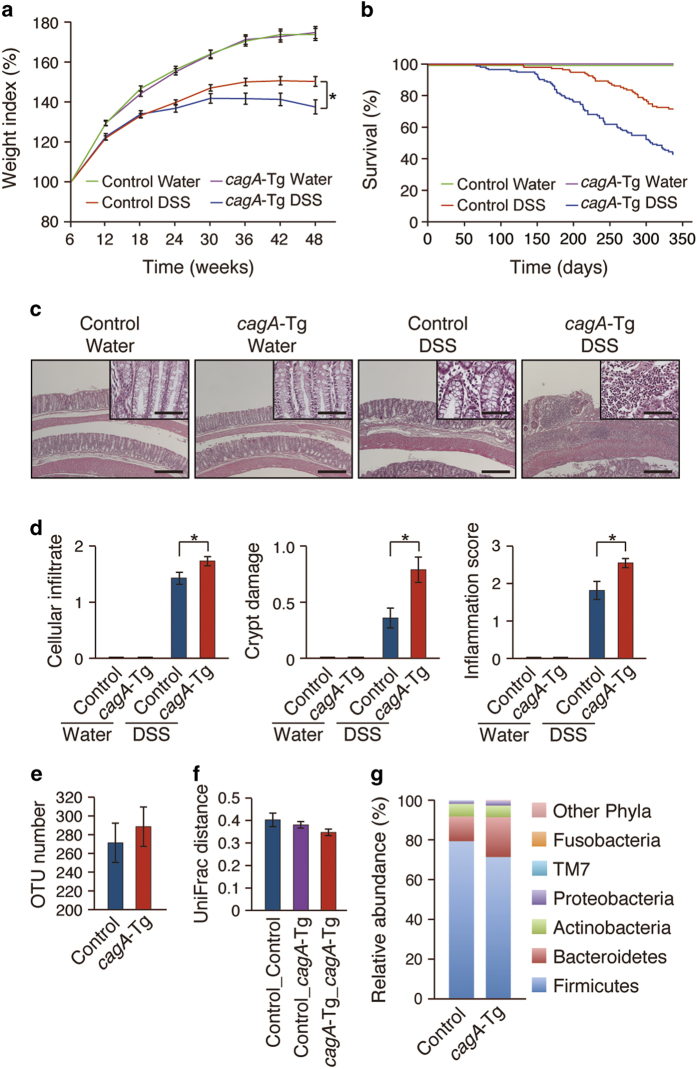
DSS-induced colitis in *cagA*-Tg mice. (a-d) The *cagA*-Tg mice and control mice were treated with or without DSS. (**a**) Body weights were measured every 6 weeks. Error bars, mean ± s.e.m. Control Water (n = 122), *cagA*-Tg Water (n = 114), control DSS (n = 91), and *cagA*-Tg DSS (n = 55). *P < 0.05 (ANOVA and Tukey test). (**b**) Kaplan-Meier survival curves. Control Water (n = 122), *cagA*-Tg Water (n = 114), control DSS (n = 91), and *cagA*-Tg DSS (n = 55). (**c**) Hematoxylin & eosin (H&E) staining of the colonic mucosa of 48-week-old *cagA*-Tg and control mice. Scale bars, 500 μm (50 μm in inlets). (**d**) Semi-quantitative analysis for the magnitude of colonic inflammation. Scoring of cellular infiltrate (left panel), crypt damage (middle panel), and inflammation (right panel) was performed as described in Materials and Methods. Error bars, mean ± s.e.m. Control Water (n = 122), *cagA*-Tg Water (n = 114), control DSS (n = 91), and *cagA*-Tg DSS (n = 47). *P < 0.05 (Student’s t-test). (**e**, **f**, **g**) Microbiome analysis of fecal samples from *cagA*-Tg (n = 6) and control (n = 6) mice. (**e**) OTU (operational taxonomic unit) number in fecal microbiota. Error bars, mean ± s.e. (n = 6). P > 0.05 (Student’s t-test). (**f**) Weighted UniFrac distance within *cagA*-Tg and control mice and between *cagA*-Tg and control mice. Error bars, mean ± s.e. (n = 6). P > 0.05 (Student’s t-test). (**g**) Microbiota compositions of *cagA*-Tg and control mice. Error bars, mean ± s.e. (n = 6). P > 0.05 (Student’s t-test).

There was no difference in the body weight curve between *cagA*-Tg and control mice without DSS administration, and none of the mice in those groups died before 48 weeks of age. Repeated administration of DSS resulted in weight loss in mice by 12 weeks of age, and DSS-treated mice exhibited greater weight loss than did non-treated mice at 42 weeks (Fig. 1a). DSS-induced physical exhaustion as determined by weight loss was more evident in *cagA*-Tg mice than in control mice (P = 0.032). Thirty-four (27.3%) of the 125 control mice and 72 (56.7%) of the 127 *cagA*-Tg mice treated with DSS died before 48 weeks of age. Kaplan-Meier survival curves confirmed a more severe clinical course for DSS-treated *cagA*-Tg mice than for DSS-treated control mice (Fig. 1b). It has been reported that male mice were more susceptible to DSS-induced colitis than female mice^16^. In the present study, DSS-treated male mice tended to die a bit earlier than DSS-treated female mice in both *cagA*-Tg and control mice. However, there was no statistically significant difference in Kaplan-Meier survival curves between male and female mice in DSS-treated *cagA*-Tg or DSS-treated control group. Autopsy of dead or dying mice during DSS treatment showed melena or enlargement of the colon due to paralytic ileus, which could become the cause of death. Because of the inflammation, colon length was decreased in DSS-treated mice compared with that in non-treated mice and was substantially shorter in DSS-treated *cagA*-Tg mice than in DSS-treated control mice (Figure S2). From these observations, we concluded that *H. pylori* CagA deteriorated DSS-induced colitis.

### Modification of DSS-induced colitis by CagA

Histological examination of fixed colonic tissues at 48 weeks of age revealed a marked thickening of the mucosal layer upon DSS treatment in both control and *cagA*-Tg mice, which was concomitantly associated with infiltration of mononuclear cells, especially macrophages/monocytes and lymphocytes (Fig. 1c, S3a, b). Loss of crypt structure, eroded epithelium, and ulceration were also observed in the colons of DSS-treated mice. Using a quantitative scoring system that weighted the severity of cell infiltration, crypt damage and mucosal/submucosal edema, we histopathologically confirmed exacerbation of DSS-induced colitis in *cagA*-Tg mice compared to that in control mice (Fig. 1d). There was no significant gender difference on severity of DSS-induced colitis in both *cagA*-Tg and control mice. Analysis of lymphocytes isolated from the colons of DSS-treated mice showed an increase in the Th17 population (Figure S3c). Expression of mRNAs for Th1 cytokines (TNFα) was also elevated in DSS-treated colonic mucosa (Figure S3d). The results were consistent with results of previous studies showing that DSS-induced colitis was associated with activation of Th1 and Th17 responses^17^,^18^,^19^,^20^.

It was possible that modification of resident microbiota in the intestinal tract of *cagA*-Tg mice enhanced DSS-induced inflammation. To test this possibility, we investigated the gut microbiome constitution in *cagA*-Tg and control C57BL/6J mice by analyzing 16S ribosomal DNA sequences from fecal samples. As a consequence, we found no significant differences in composition of the intestinal microbiome between the two mouse groups (Fig. 1e, f, g). The result argues against the idea that exacerbation of DSS-induced colitis in *cagA*-Tg mice was due to altered composition of gut microbiota.

### Reduced IκB pool in the alimentary tract of *cagA*-Tg mice

**Figure 2:**
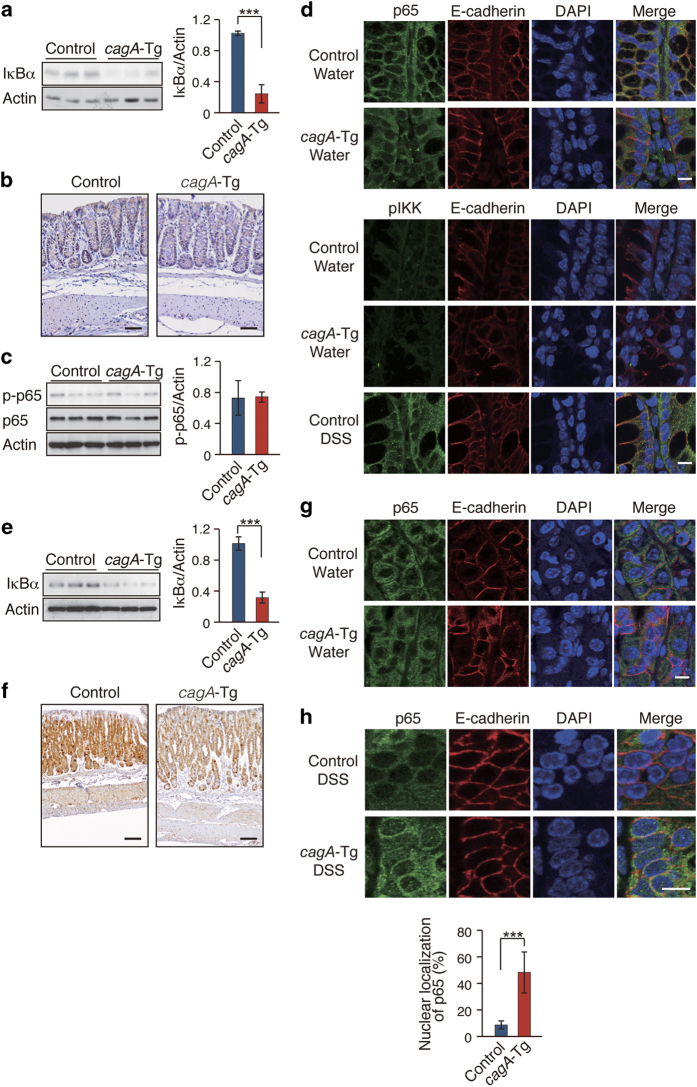
Effect of CagA expression on NF-κB signaling in mice. (**a**) Immunoblot analysis of IκBα in the colon of 48-week-old *cagA*-Tg and control mice. Lysates prepared from the colon were immunoblotted with the indicated antibodies (left panel). Quantification of the intensity of IκBα relative to actin (right panel). Error bars, mean ± s.d. (n = 3). ***P < 0.001 (Student’s t-test). (**b**) Immunostaining of IκBα in the colonic mucosa from *cagA*-Tg mice or control mice without DSS treatment. Scale bars, 50 μm (**c**) Immunoblot analysis of phosphorylated p65 (p-p65) in the colon of 48-week-old *cagA*-Tg and control mice (left panel). Quantification of the intensity of p-p65 relative to actin (right panel). Error bars, mean ± s.d. (n = 3). (**d**) Immunostaining of p65 (upper panel) and phosphorylated IKKα/β (pIKK) (lower panel) in the colonic mucosa of 48-week-old *cagA*-Tg or control mice. Nuclei were visualized by DAPI. Scale bars, 10 μm. (**e**) Immunoblot analysis of IκBα in the stomach of 48-week-old *cagA***-**Tg and control mice (left panel). Quantification of the intensity of IκBα relative to actin (right panel). Error bars, mean ± s.d. (n = 3). ***P < 0.001 (Student’s t-test). (**f**) Immunostaining of IκBα in the stomach from *cagA*-Tg mice or control mice without DSS treatment. Scale bars, 100 μm. (**g**) Immunostaining of p65 in the stomach from *cagA*-Tg mice or control mice without DSS treatment. Nuclei were visualized by DAPI. Scale bar, 10 μm. (**h**) Immunostaining of p65 in the colonic mucosa from *cagA*-Tg mice or control mice with DSS treatment. Scale bar, 10 μm (upper panel). Percentage of cells showing nuclear localization of p65 (lower panel). Error bars, mean ± s.d. (n = 6). ***P < 0.001 (Student’s t-test).

To determine whether CagA-mediated enhancement of colitis involves the signaling pathway that activates NF-κB, we compared the levels of IκBα, a major isoform of the NF-κB inhibitor IκB, in *cagA*-Tg mice and control mice. Anti-IκBα immunoblotting revealed that the level of IκBα was significantly reduced in the colon of *cagA*-Tg mice compared to that in the colon of control mice (Fig. 2a). Immunohistochemical analysis confirmed reduced expression of IκBα in colonic epithelial cells in both crypt and villus compartments in *cagA*-Tg mice (Fig. 2b). Because of low levels of IκBα expression in mesenchymal and smooth muscular cells, epithelial cells were thought to be primarily responsible for the reduction of IκBα in the colon. Despite reduced IκBα, however, there was no difference in the level of active NF-κB (phosphorylated p65/RelA) between the two mouse groups (Fig. 2c). Indeed, the p65/RelA component of NF-κB was almost exclusively present in the cytoplasm of colonic epithelial cells in both *cagA*-Tg and control mice (Fig. 2d, upper panels). Thus, the reduced level of IκBα in *cagA*-Tg mice on its own was insufficient to activate NF-κB. Since the levels of the active form of IκB kinase (pIKK) were comparable in *cagA*-Tg and control mice (Fig. 2d, lower panels), the IκBα reduction in *cagA*-Tg mice was unlikely to be due to accelerated degradation of IκB by IKK. Reduced IκB was also observed in the stomach mucosa of *cagA*-Tg mice (Fig. 2e,f), although, again, it did not cause nuclear translocalization of NF-κB (Fig. 2g). Treatment of mice with DSS, however, gave rise to the detection of colonic epithelial cells with nuclear NF-κB localization, the incidence and degree of which were greater in *cagA*-Tg mice than in control mice (Fig. 2h). The observations may explain why *cagA*-Tg mice did not spontaneously develop colonic inflammation but showed deterioration of DSS-induced colitis.

In contrast to the gastrointestinal epithelial cells, expression levels of IκBα in peritoneal macrophages were the same in *cagA*-Tg and control mice. Also, there was no difference in the magnitude of nuclear translocalization of NF-κB in response to LPS between macrophages isolated from *cagA*-Tg mice and those isolated from control mice (Figure S4). Thus, reduction of IκBα in *cagA*-Tg mice was relatively specific to the gastrointestinal epithelial cells, possibly due to differences in the levels of CagA expression in different tissues.

### Oncogenic cooperation between CagA and DSS

Repeated administration of DSS gave rise to macroscopically recognizable polypoid lesions in 11% of the control mice that were alive at 48 weeks of age (10/91 mice) (Figure S5a). No such polyps were found in age-matched *cagA*-Tg mice (0/114) or control mice (0/122) without DSS treatment. The incidence of polypoid lesions in the colon was elevated in *cagA-*Tg mice that had survived DSS treatment compared to that in DSS-treated control mice (Figure S5b). However, multiplicity and size of the polyps induced by DSS were not significantly different between *cagA*-Tg mice and control mice (Figure S5b).

**Figure 3:**
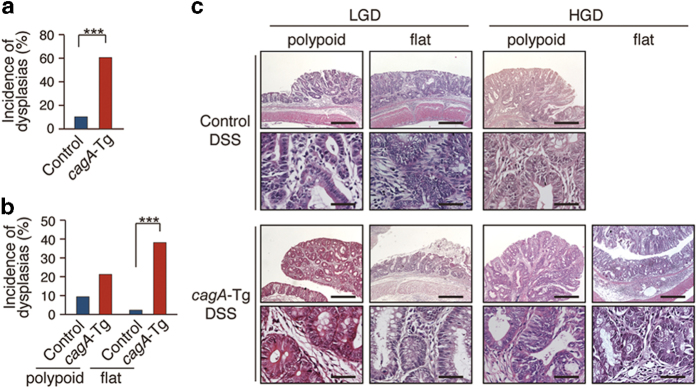
Histological analysis of DSS-induced dysplasias. (**a**) Incidence of dysplasias developed in *cagA*-Tg mice or control mice with DSS treatment. Control (n = 91) and *cagA*-Tg (n = 47). ***P < 0.001 (Mann-Whitney U test). (**b**) Quantitative analysis of polypoid and flat dysplasias developed in *cagA*-Tg mice or control mice with DSS treatment. Control (n = 91) and *cagA*-Tg (n = 47). ***P < 0.001 (Mann-Whitney U test). (**c**) H&E staining of the low-grade dysplasias (LGD) and high-grade dysplasias (HGD) developed in the colonic mucosa of *cagA*-Tg mice or control mice with DSS treatment. Scale bars, 500 μm in the zoomed-out images (upper panels), 50 μm in the zoomed-in images (lower panels).

Development of neoplasia (dysplasia or carcinoma) in the colon of mice that survived the DSS treatment at 48 weeks of age was next examined by microscopy (Table 1). Ten dysplasias and 28 dysplasias were found in 91 DSS-treated control mice and 47 DSS-treated *cagA*-Tg mice, respectively (Fig. 3a). The mean numbers of dysplasias per mouse were 0 (non-treated control mice and non-treated *cagA*-Tg mice), 1.13 (DSS-treated control mice), and 2.15 (DSS-treated *cagA*-Tg mice). In DSS-treated control mice, types of dysplasias were 80% polypoid (8/10) and 20% flat (2/10). In contrast, 36% (10/28) of the dysplasias were polypoid and 64% (18/28) were flat in DSS-treated *cagA*-Tg mice (Fig. 3b,c and Table 1). The results indicated that CagA robustly induced flat dysplasia in conjunction with DSS. Among the 28 dysplasias found in DSS-treated *cagA*-Tg mice, 24 were low-grade dysplasias (LGDs) (polypoid : flat = 7 : 17) and 4 were high-grade dysplasias (HGDs = carcinomas *in situ*) (polypoid : flat = 3 : 1). Of the 10 dysplasias found in DSS-treated control mice, 8 were LGDs (polypoid : flat = 6 : 2) and 2 were HGDs (polypoid : flat = 2 : 0). Accordingly, CagA contributed to the development of dysplasia but did not accelerate progression of the grade of dysplasia from LGD to HGD. The results are consistent with the notion that CagA acts in the early phases but not late phases of carcinogenesis^21^. Based on these observations, we concluded that CagA promoted the development of colonic neoplasia, especially flat dysplasia, in the DSS-inflamed colonic mucosa.

### Oncogenic changes associated with colonic dysplasias

**Figure 4:**
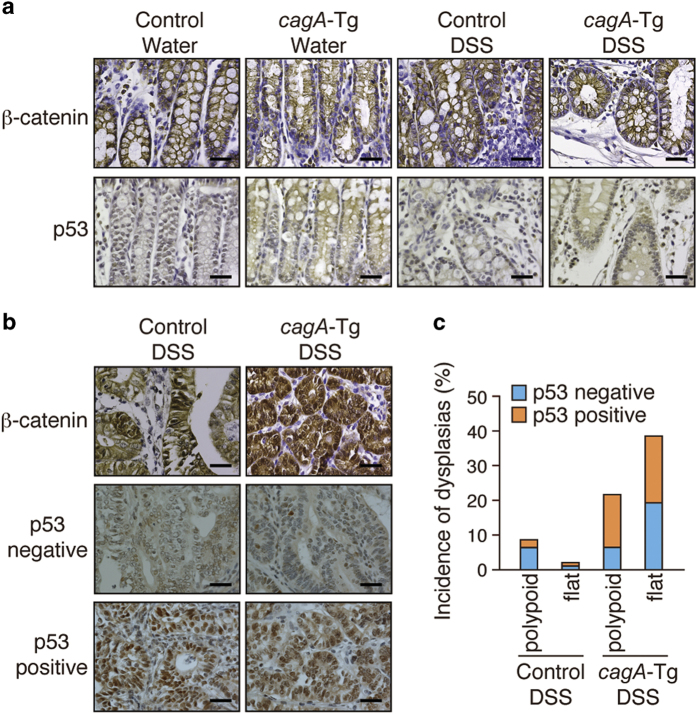
**Immunostaining of β-catenin and p53 in DSS-induced dysplasias.** (**a**) Immunostaining of β-catenin and p53 in the colonic mucosa of *cagA-*Tg or control mice with or without DSS treatment. Scale bars, 40 μm. (**b**) Immunostaining for β-catenin and p53 in dysplastic lesions of the colon. In the p53-immunostaining samples, both nuclear p53 staining-negative and -positive cases are shown. Scale bars, 40 μm. (**c**) Quantitative analysis of p53-negative and p53-positive dysplasias in DSS-treated *cagA-*Tg (n = 47) and control (n = 91) mice.

To gain insights into the mechanism underlying the development of dysplastic lesions in the colon, we examined the status of the canonical Wnt pathway and that of p53, which are perturbed in many gastrointestinal neoplasias^22^. Nuclear accumulation of β-catenin indicates activation of the canonical Wnt signal. Although DSS treatment did not induce nuclear β-catenin accumulation in the colon of *cagA*-Tg or control mice (Fig. 4a), strong nuclear staining of β-catenin was observed in all of the dysplastic lesions that developed in *cagA*-Tg or control mice (Fig. 4b). Thus, deregulation of the canonical Wnt signal was a common feature of DSS-induced colonic dysplasias. We next examined the status of p53. Wild-type p53 has a short half-life, making it hardly detectable by immunostaining. In contrast, missense p53 mutants often display a much longer half-life and therefore exhibit strong nuclear accumulation^23^. Whereas wild-type p53 is stabilized in cells undergoing DNA damage^24^ or receiving innate immune signaling^25^, strong nuclear staining of p53 is generally interpreted as indicating a p53 mutation. Among the 10 polypoid dysplasias found in *cagA*-Tg mice, 7 showed strong nuclear staining of p53 and 3 did not. Likewise, 9 of the 18 flat dysplasias that developed in *cagA*-Tg mice were positive for nuclear p53 staining (Fig. 4c and Table 1). In contrast, 6 of the 8 polypoid dysplasias that developed in DSS-treated control mice were negative for p53 staining. Accordingly, the incidence of dysplasias (polypoid + flat) with abnormal p53 staining was significantly greater in DSS-treated *cagA*-Tg mice (16/28) than in DSS-treated control mice (3/10). Importantly, nuclear accumulation of p53 was strictly limited to the dysplastic lesions and was not observed in other parts of the intestinal mucosa of DSS-treated *cagA-*Tg or control mice (Fig. 4a,b). This observation argues strongly against the idea that the p53 staining was due to genotoxic stress^24^ or innate immune activation^25^, which should induce nuclear accumulation of wild-type p53 diffusely in the DSS-inflamed colonic mucosa, not restricted to dysplasias. Hence, colonic dysplasias that developed in DSS-treated *cagA*-Tg mice were characterized by deregulated canonical Wnt signal and were frequently associated with p53 mutation.

### Mechanistic and functional insights into CagA-mediated IκB reduction

**Figure 5:**
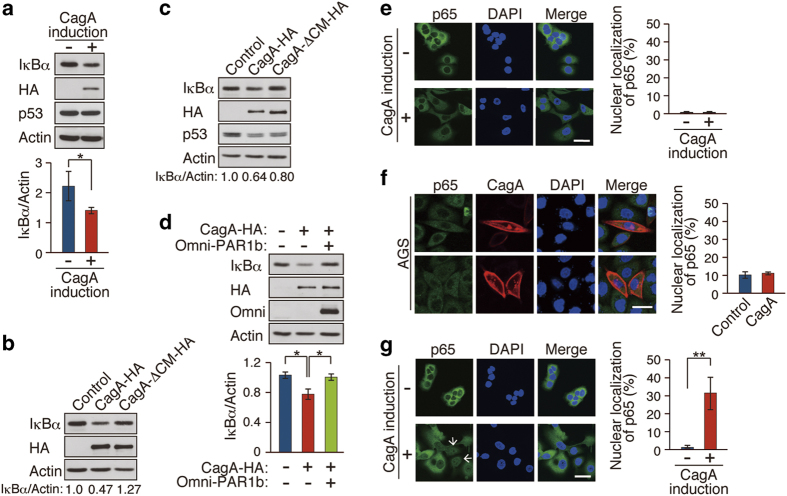
Mechanistic and functional insights into CagA-mediated IκB reduction. (**a**) WT-A10 cells were induced to express HA-tagged CagA by Dox depletion for 5 days. Lysates prepared were immunoblotted with the indicated antibodies (upper panel). Quantification of the intensity of IκBα relative to actin (lower panel). Error bars, mean ± s.d. (n = 3). *P < 0.05 (Student’s t-test). (**b**) MKN28 cells were infected with lentiviruses for 6 days at a multiplicity of infection of 27. Lysates prepared were subjected to immunoblot analysis. Relative amount of IκBα was calculated from the immunoblotting data. (**c**) GES-1 cells were infected with lentiviruses for 6 days at a multiplicity of infection of 27. Lysates prepared were subjected to immunoblot analysis. Relative amount of IκBα was calculated from the immunoblotting data. (**d**) WT-A10 cells were induced to express HA-tagged CagA by Dox depletion. At 12 h after induction, cells were transiently transfected with an Omni-PAR1b vector for 4 days. Lysates prepared were subjected to immunoblot analysis (upper panel). Quantification of the intensity of IκBα relative to actin (lower panel). Error bars, mean ± s.d. (n = 3). *P < 0.05 (Student’s t-test). (**e**) Immunostaining of p65 in WT-A10 cells with or without CagA induction for 5 days. Nuclei were visualized by DAPI. Scale bar, 40 μm (left panel). Percentage of cells showing nuclear localization of p65 (right panel). Error bars, mean ± s.d. (n = 3). (**f**) Immunostaining of p65 in AGS cells transiently transfected with a CagA-HA vector for 24 h. Scale bar, 40 μm (left panel). Percentage of cells showing nuclear localization of p65 (right panel). Error bars, mean ± s.d. (n = 3). (**g**) WT-A10 cells were induced to express CagA for 5 days, followed by TNFα (1 ng/ml) treatment for 20 min. Cells showing nuclear p65 staining are indicated by white arrows. Scale bar, 40 μm (left panel). Percentage of cells showing nuclear localization of p65 (right panel). Error bars, mean ± s.d. (n = 3). **P < 0.01 (Student’s t-test).

To investigate the mechanism by which CagA reduces the cellular IκBα pool, we made use of MKN28-derived gastric epithelial cells (WT-A10 cells), which inducibly express wild-type CagA by the *tet-off* system^26^. Sustained induction of CagA in WT-A10 cells (more than 5 days) gave rise to a reduction in the level of IκB (Fig. 5a and S6a), reproducing the *in vivo* effect of CagA on IκB. Treatment of WT-A10 cells with the Src family kinase inhibitor PP2 did not prevent CagA-mediated reduction of IκB (Figure S6b), indicating that the CagA activity was independent of CagA tyrosine phosphorylation. Since CagA interacts with PAR1 independently of tyrosine phosphorylation^5^, we hypothesized that PAR1 is involved in the reduction of IκB by CagA. To test this idea, MKN28 cells or GES-1 gastric epithelial cells were infected with a lentivirus carrying the wild-type *cagA* gene or its mutant encoding CagA that cannot interact with PAR1 (CagA-ΔCM). In contrast to wild-type CagA, CagA-ΔCM was incapable of reducing IκB (Fig. 5b,c). Given this, we next inhibited the expression of endogenous PAR1b, a major isoform of PAR1 in epithelial cells, by siRNA. As expected, PAR1 knockdown decreased the level of IκB like CagA (Figure S6c). Reciprocally, ectopic expression of PAR1b counteracted the ability of CagA to reduce IκB (Fig. 5d). These observations indicated that the CagA-PAR1 interaction plays a key role in reduction of the level of IκB by CagA.

As was observed in *cagA*-Tg mice, CagA-mediated reduction of IκB in WT-A10 cells failed to promote translocalization of NF-κB to the nucleus whereas treatment with the proinflammatory cytokine TNFα (20 ng/ml) elicited strong nuclear accumulation of NF-κB (Figure 5e and S6d). Likewise, transient expression of CagA in AGS gastric epithelial cells, which induced cell elongation known as the hummingbird phenotype^4^, did not provoke nuclear translocation of NF-κB (Fig. 5f). Given this, we sought to investigate whether CagA-mediated IκB reduction increases the cellular sensitivity to an extrinsic cue that stimulates NF-κB. To do so, we treated WT-A10 cells with a suboptimal dose of TNFα (1 ng/ml), the amount of which did not induce nuclear localization of NF-κB. The weak TNFα stimulation was capable of eliciting nuclear translocalization of NF-κB only when CagA was present, indicating that CagA made cells hypersensitive to stimuli that activate NF-κB (Fig. 5g and S6d). Enhanced cellular sensitivity to stimuli that acivate NF-κB may contribute to survival and expansion of CagA-expressing precancerous cells that underlie polypoid dysplasia formation.

It has recently been reported that CagA promotes degradation of p53 by forming a complex with Apoptosis-stimulating protein of p53-2 (ASPP2)^27^. Consistent with this, we found that ectopic expression of CagA in GES-1 gastric epithelial cells, which carry wild-type p53, caused a reduction in the level of p53 (Fig. 5c). In contrast, CagA failed to decrease the p53 level in MKN28 cells, which carry a mutant p53 protein (Fig. 5a). Since mutant p53 does not bind to ASPP2^28^, the CagA-ASPP2 complex may fail to promote degradation of mutant p53. The notion explains accumulation of p53 in dysplasic lesions developed in *cagA*-Tg mice (see also DISCUSSION).

### CagA-mediated activation of inflammasomes

**Figure 6:**
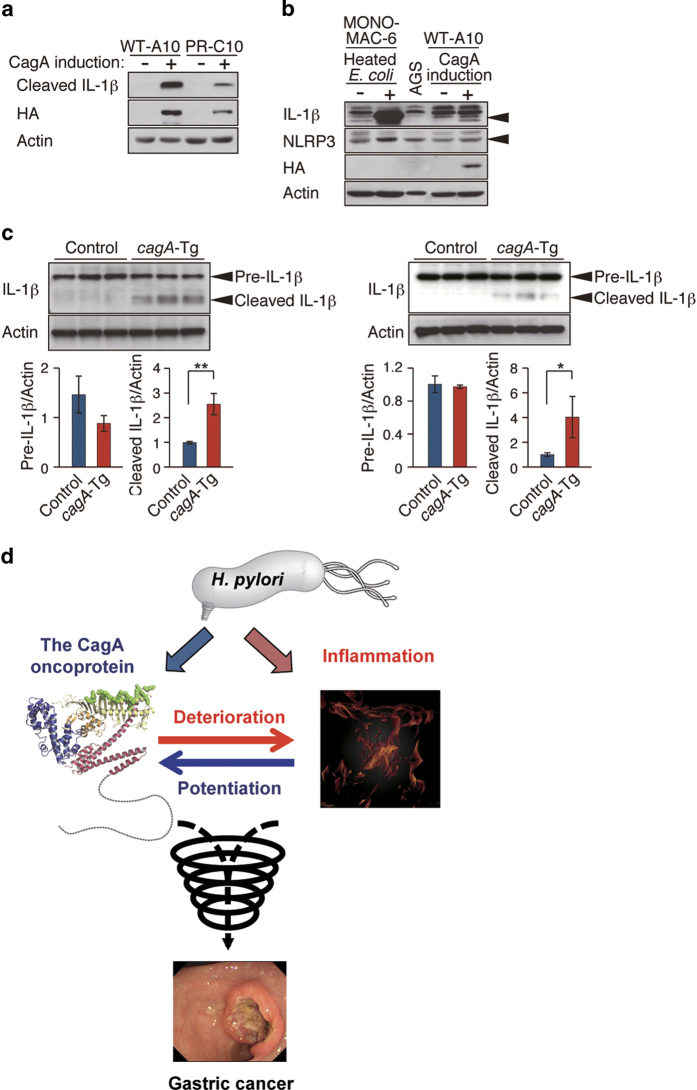
Effect of CagA on inflammasomes in epithelial cells. (**a**) Immunoblot analysis of mature IL-1*β* (cleaved IL-1β) in WT-A10 and PR-C10 cells cultured with or without Dox for 6 days. (**b**) Immunoblot analysis of IL-1β and NLRP3 in MONO-MAC-6, AGS, and WT-A10 cells. MONO-MAC-6 cells were incubated with killed *E. coli* for 24 h. (**c**) Immunoblot analysis of IL-1β in the colon (left upper panel) and stomach (right upper panel) of 48-week-old *cagA*-Tg and control mice. Quantification of the intensity of precursor and mature IL-1β relative to actin (lower panels). Error bars, mean ± s.d. (n = 3). *P < 0.05, **P < 0.01 (Student’s t-test). (**d**) A proposed model based on the results of the current study. CagA and inflammation reinforce each other and thereby trigger a downward spiral that accelerates neoplastic transformation.

During the course of the experiment using MKN28-derived WT-A10 cells, we noticed that long-term induction of CagA not only reduced IκB but also gave rise to secretion of the mature form of IL-1β, which was never produced without CagA induction (Fig. 6a). Production of mature IL-1β, which requires activation of inflammasomes, was independent of CagA tyrosine phosphorylation because it was observed upon inducible expression of a phosphorylation-resistant CagA in MKN28-derived PR-C10 cells (Fig. 6a)^26^. To determine whether the observed inflammasome activation is specific to CagA or is due to non-specific overexpression of an ectopic protein, we employed MKN28-derived cells that inducibly express CDX1, an intestinal-specific transcription factor that is unrelated to CagA, using the tet system like WT-A10 cells^29^. As a result, CDX1 overexpression did not convert pro-IL-1β to mature IL-1β in MKN28 cells (Figure S7a). Since CagA induction *per se* did not cause nuclear translocalization of NF-κB, it was unlikely that CagA primed inflammasomes via NF-κB activation. Given that non-immune cells, including epithelial cells, also express Nod-like receptors (NLRPs) as well as IL-1β^30^,^31^, it was possible that inflammasomes were specifically assembled and activated in epithelial cells by CagA. In fact, NLRP3, a Nod-like receptor component that constitutes NLRP3 inflammasomes, was expressed in WT-A10 cells (Fig. 6b). Additionally, both IL-1β mRNA and precursor IL-1β protein were present, albeit at low levels, in gastric epithelial cells (Fig. 6b and S7b). The precursor form of IL-1β was also detectable in the colon and stomach mucosae of both *cagA*-Tg mice and control mice, whereas the mature form of IL-1β was specifically detectable in the colon and stomach of *cagA*-Tg mice (Fig. 6c). We therefore concluded that CagA causes inflammasome activation independently of the NF-κB signaling pathway, which may further contribute to the deterioration of DSS-induced colitis.

## Discussion

The present study revealed a symbiotic relationship between the *H. pylori* CagA oncoprotein and inflammation in pathogenesis. The proinflammatory role of CagA is consistent with clinico-pathological observations as well as results of animal infection studies showing that *cagA*-positive *H. pylori* strains elicit more severe mucosal inflammation than do *cagA*-negative strains^32^,^33^,^34^. Mechanistically, CagA diminishes the level of IκB, an inhibitor of NF-κB. However, the CagA-mediated IκB reduction is insufficient to automatically elicit nuclear translocalization and subsequent activation of NF-κB. Our observations support the results of previous studies indicating that CagA *per se* is not a potent inflammogen^35^,^36^. Paradoxically, however, several reports have also shown direct activation of NF-κB by CagA^37^,^38^,^39^. These confusing observations may be reconciled at least partly by the results of the present work demonstrating modest downregulation of IκB by CagA and thus, depending on the level of IκB as well as the amount of CagA delivered in a given cellular setting, the bacterial protein could activate NF-κB directly or only in the context of an additional signal(s) that cross-reacts with the NF-κB signaling pathway. Since *H. pylori* is capable of activating NF-κB independently of CagA^11^,^12^,^13^, a primary role of CagA in inflammation may be to amplify the magnitude of NF-κB activation at the site of *H. pylori* infection. Reduction of IκB requires interaction of CagA with the serine/threonine kinase PAR1, which regulates microtubule stability by phosphorylating microtubule-associated proteins (MAPs)^40^. Since microtubule destabilization leads to the activation of NF-κB by promoting IκBα degradation^41^,^42^, impairment of the microtubule system by CagA-PAR1 interaction may give a cytoskeletal cue that stimulates IκB degradation.

In DSS-induced colitis model, stimulation of TLRs, especially TLR2 and TLR4, by commensal bacteria plays an important role in the pathogenesis^43^,^44^. Notably, however, steady-state activation of TLRs by the gut microbiota is also required to maintain the intestinal architectural integrity and to repair intestinal surface injuries^45^. When DSS-induced epithelial injury overwhelms the repair activity, disrupted epithelial barriers promote hyperactivation of TLR signaling that induces robust inflammatory responses. Contribution of innate immune responses of epithelial cells in DSS-induced colitis has been corroborated by the finding that the colitis is deteriorated in transgenic mice specifically expressing a constitutively activated form of IKK in intestinal epithelial cells^46^. More recently, mutant p53 was found to promote DSS-induced colitis by prolonging NF-κB activation in intestinal epithelial cells^47^. Since the *cagA* gene was driven by the *β-actin* promoter in *cagA*-Tg mice, we cannot exclude the possibility that CagA expressed in immune cells rather than CagA expressed in epithelial cells renders the host hypersensitive to inflammogenic insults. However, we previously reported that bone marrow-derived dendritic cells isolated from *cagA*-Tg mice were hypo-reactive to LPS^48^. Also, there was no difference in the responses of peritoneal macrophages toward LPS between *cagA*-Tg mice and control mice. We therefore consider that hyperresponsiveness of colonic epithelial cells to stimuli that activate NF-κB contributes substantially to the deterioration of DSS-induced colitis in *cagA*-Tg mice.

In DSS-induced colitis, the cytokine profile is skewed toward Th1/Th17 responses^17^,^18^,^19^, although Th2 response is also elevated in its chronic phase^17^,^20^. Consistently, Th1 response was stimulated and the Th17 population was increased in chronic colitis induced by DSS in both *cagA*-Tg and control mice. Transgenic insulin-gastrin (INS-GAS) mice have high circular gastrin level and show spontaneous development of gastric neoplasia. Unlike *cagA*-Tg mice, however, INS-GAS mice progressively develop gastritis, atrophy, and dysplasia/carcinoma as in human gastric cancer cases^49^. Under germ-free conditions, INS-GAS mice exhibit delayed onset of gastric tumor development. The delay of tumor progression was partly recovered by *H. pylori* monoassociation and further restored by additional colonization of gut microbiota in the hypochlorhydric stomach. This bacterial infection-accelerated tumorigenesis in INS-GAS mice was concomitantly associated with elevated Th1- and Th17-dependent inflammatory responses^50^. In contrast, no overt difference was found in the composition of intestinal microbiota between *cagA*-Tg and control mice. Accordingly, the pathogenesis of INS-GAS mice may be substantially different from that of DSS-treated *cagA*-Tg/DSS mice. Nevertheless, both animal models point to the importance of Th1- and Th17-mediated immune responses in the development of neoplasias.

CagA activates inflammasomes in epithelial cells independently of NF-κB signaling. Although the underlying mechanism remains unclear, several viral proteins have been reported to stimulate inflammasomes via a disordered protein structure^51^. Given that the C-terminal CagA is characterized by the extensive structural disorder^52^, a certain type of inflammasome might recognize the disordered CagA tail as a danger signal. The functional relationship between CagA and inflammasome is of particular interest in light of the fact that polymorphisms in the *IL1B* gene, which encodes IL-1β, are associated with increased gastric cancer risk in the context of *H. pylori* infection^53^ and the fact that transgenic overexpression of IL-1β in parietal cells induces gastric dysplasia/carcinoma in mice^54^. The CagA-mediated inflammasome activation may provide a mechanistic link between *H. pylori* CagA and IL-1β in the development of gastric cancer.

In humans, both flat and polypoid dysplasias are associated with inflammatory bowel disease (IBD)^55^. Mice treated with DSS also develop polypoid and flat dysplasias, the relative frequencies of which are influenced by genetic background^15^. In the DSS colitis model, Wnt deregulation is an initial event and p53 inactivation is a late event in polypoid dysplasia formation. In contrast, p53 inactivation is an early change, whereas Wnt deregulation is a relatively late manifestation in the development of flat dysplasia^15^,^56^. Thus, aberrant Wnt activation and impaired p53 function are both associated with dysplastic changes, in which the relative order and magnitude of Wnt deregulation and p53 inhibition may determine the histological type of dysplasia. In the present study, the development of dysplasias was markedly enhanced in *cagA*-Tg mice treated with DSS. Since CagA deregulates canonical Wnt signaling through multiple distinct mechanisms^21^, the bacterial protein may stimulate dysplasia formation by aberrantly intensifying the Wnt signal in the inflammatory micro-milieu. A unique observation here is that CagA markedly potentiates *de novo* formation of flat dysplasia, which was rarely observed in DSS-treated control mice. Considering early inactivation of p53 during flat dysplasia formation by DSS^56^, malfunctioning of p53 in colonic epithelial cells expressing CagA may underlie the increased flat dysplasias in DSS-treated *cag*A-Tg mice. Consistent with this idea, flat dysplasias that developed in *cagA*-Tg mice exhibited strong nuclear staining of p53, a distinct feature of mutant p53. At first glance, this observation seemed to be inconsistent with the results of a previous study showing that CagA stimulates p53 degradation by interacting with ASPP2^27^. However, we found in this work that CagA does not promote degradation of mutant p53, probably because mutant p53 does not bind to ASPP2^28^. This finding provides additional support for the notion that p53 that accumulated in the colonic dysplasias was a mutated from. Indeed, CagA is capable of promoting mutation of the *p53* gene through aberrant induction of activation-induced cytidine deaminase (AID), which is the transcriptional target of NF-κB^57^. More recently, mutant p53 in colonic epithelial cells has been shown to potentiate DSS-induced colitis and to promote colitis-associated carcinogenesis by prolonging NF-κB activation^47^. Those findings are very similar to those of the present work, raising the possibility of a functional similarity between CagA and mutant p53 in promoting carcinogenesis. Highly activated NF-κB should sustain oncogenic action of CagA by protecting CagA-expressing cells from apoptosis.

The *cagA*-Tg mice provide a unique opportunity that enabled us to investigate pathophysiological interplay between the *H. pylori* CagA oncoprotein and inflammation *in vivo*. A mutual functional reinforcement between CagA and inflammation generates a downward “carcinogenic spiral” that accelerates neoplastic transformation of epithelial cells (Fig. 6d). The present work points to the notion that *H. pylori* CagA predisposes the host to inflammation, which in turn creates an environment favorable for CagA-mediated carcinogenesis. Amelioration of *H. pylori*-induced inflammation should effectively prevent the development of gastric cancer by dampening the oncogenic action of CagA.

## Methods

### Animals

Generation of *cagA*-transgenic mice (*cagA*-Tg mice) has been described previously^6^. The *cagA*-Tg mice expressed relatively high levels of *cagA* mRNA in colon among various tissues examined. The *cagA*-Tg mice were maintained by crossing C57BL/6J mice (CLEA JAPAN). For DSS studies, *cagA*-Tg mice and the littermates housed in same cages after splitting into male and female, provided sterile rodent diet and water *ad libitum*. The animal room was quarantined by air flow and kept at a constant temperature and humidity under specific pathogen-free (SPF) conditions. Microbiological monitoring was performed every three month. Only *Trichomonas muris* was positive and other specific pathogens including *Helicobacter bilis* and *Helicobacter hepaticus* were negative. *Trichomonas muris* is an intestinal protozoa that has been considered a commensal agent not related to alterations of the animal health or interferences in experimental results^58^. All animal experiments were approved by ethics committee of the Graduate School of Medicine, the University of Tokyo and were performed according the guidelines for care and use of laboratory animals of the University of Tokyo.

### Antibodies

Anti-IκBα (L35A5), anti-IκBα (C21), anti-p65 (D14E12), anti-phospho-IKKα/β (S176/180, 16A6) and anti-cleaved IL-1β (D116) antibodies were from Cell signaling Technology; anti-NLRP3 (nalpy3-b, ab17267) and anti-IL-1β (ab9722) antibodies were from abcam; anti-Omni (M-21), anti-p53 (FL393), and anti-Actin (C-11) antibodies were from Santa Cruz Biotechnology; anti-p53 antibody was from Dako; anti-β-catenin antibody was from BD Biosciences; anti-HA (3F10) antibody was from Roche.

### Cell culture and transfection

MKN28 and AGS human gastric epithelial cells were cultured as previously described^5^,^26^. GES-1 human gastric epithelial cells were cultured in Dulbecco’s modified Eagle’s medium (DMEM) supplemented with 10% fetal bovine serum (FBS). MONO-MAC-6 human monocytes were cultured in RPMI 1640 medium supplemented with 10% FBS and human insulin (10 μg/ml). WT-A10 and PR-C10 cells are MKN28-derived stable transfectant clones that inducibly express wild-type CagA and phosphorylation-resistant CagA, respectively, using a *tet-off* system^26^. MKN28 and WT-A10 cells were transfected with plasmids using Lipofectamine and Plus reagents (Invitrogen). AGS cells were transfected with plasmids using FuGENE 6 transfection reagent (Promega).

### Expression vectors

Mammalian expression vectors for hemagglutinin (HA)-tagged CagA (*H. pylori* NCTC11637 strain-derived CagA) and Omni-tagged PAR1b have been described previously^5^. Recombinant lentiviruses that express HA-tagged ABD-type East Asian CagA (CagA-HA) and the CagA mutant lacking the CM sequence (CagA-ΔCM-HA) were generated using Lentivector Expression Systems (System Biosciences).

### Induction of colitis

Six-week-old mice were administered 2% DSS (MPBiomedicals, MW36000-55000) in drinking water for 4 days, followed by normal drinking water for 17 days. This DSS administration was repeated for 14 cycles.

### Clinical evaluation of the colon

Body weight and survival rate were determined. The body weight index was determined every 6 weeks by the following equation: 100 x (body weight at the *x-*th day)/(body weight before DSS administration).

### Histopathological evaluation

At 48 weeks of age, mice were killed via inhalation euthanasia with carbon dioxide and entire colon was excised to measure the colon length. Colons incised longitudinally were washed with phosphate-buffered saline (PBS) and fixed in 10% formaldehyde buffer. The colons were rolled from the distal to proximal end as Swiss roll form and embedded in paraffin. Tissue sections were stained with hematoxylin and eosin (H&E). Histology was scored in a blinded fashion as described previously^59^. Briefly, the inflammation scores were determined as a combination of inflammatory cell filtration (score 0-3) and crypt damage (score 0-3). For inflammatory cell filtration, the filtration of occasional inflammatory cells in the lamina propria was scored as 0, increased numbers on inflammatory cells in the lamina propria was scored as 1, confluence of inflammatory cells extending into submucosa was scored as 2, and inflammatory cells extending into the layers of serous membrane was scored 3. For crypt damage, no mucosal damage was scored as 0, the presence of lymphoepithelial lesions was scored as 1, mucosal surface erosion or focal ulceration was scored as 2, and severe mucosal damage and extension into deeper structures of the bowel wall were scored as 3.

### Immunocytochemistry

For immunohistochemistry, formalin-fixed paraffin-embedded tissues were sliced into 4-mm-thick sections. Slides were deparafinized with xylene and heated for 15 min in citrate buffer (pH 6.0) using microwave. Endogenous peroxidase activity was blocked with 0.3% H_2_O_2_ in methanol and then non-specific binding was blocked with 10% bovine serum albumin (BSA) in Tris-buffered saline containing 0.1% Tween 20 (TBST) or mouse on mouse kit (VECTOR). The slides were incubated with primary antibodies and then incubated with appropriate secondary antibodies. Reacted antibodies were detected using ABC Elite kit (VECTOR) and diaminobenzidine (DAB) (VECTOR). Immunostaining of cultured cells was performed as described previously^5^. Fluorescent images were obtained using TCS-SPE (Leica) and FLUOVIEW FV1200 (Olympus) confocal microscope systems.

### Immunoblotting

Tissue homogenates were lysed in the lysis buffer (50 mM Tris-HCl, pH 7.5, 100 mM NaCl, 5 mM EDTA, 1% Triton X-100, 10% glycerol, 2 mM Na_3_VO_4_, 10 mM NaF, 10 mM β-glycerophosphate, 10 μg/ml aprotinin, 10 μg/ml leupeptin, 10 μg/ml trypsin inhibitor, and 2 mM phenylmethylsulfonyl fluoride). Cultured cells were harvested and lysed in the lysis buffer (50 mM Tris-HCl, pH 7.5, 100 mM NaCl, 5 mM EDTA, 0.5% NP-40, 2 mM Na_3_VO_4_, 10 mM NaF, 10 mM β-glycerophosphate, 10 μg/ml aprotinin, 10 μg/ml leupeptin, 10 μg/ml trypsin inhibitor, and 2 mM phenylmethylsulfonyl fluoride). Lysates were resolved by SDS-polyacrylamide gel electrophoresis (PAGE) and subjected to immunoblotting. Proteins were visualized using western blot chemiluminescence reagent (PerkinElmer Life Sciences). Intensity of chemiluminescence was quantified using the LAS4000 system (FUJIFILM).

### Microbiome analysis

Fecal samples collected from *cagA*-Tg mice (40.3 ± 8.09 weeks of age, n = 6) and age-matched control mice (41.8 ± 5.38 weeks of age, n = 6) were immediately frozen in liquid nitrogen and stored at –80°C until for use. Bacterial 16S rRNA amplicon sequencing and analysis were performed as previously described^60^.

### Statistical analysis

Data were statistically analyzed by Student’s t-test, Mann-Whitney U test, Tukey test, and ANOVA. Kaplan-Meier analysis was used for comparison of mouse survival. P < 0.05 was considered to be statistically significant.

## Additional Information

**How to cite this article**: Suzuki, N. *et al*. Mutual reinforcement of inflammation and carcinogenesis by the *Helicobacter pylori* CagA oncoprotein. *Sci. Rep.* **5**, 10024; doi: 10.1038/srep10024 (2015).

## Supplementary Material

Supplementary Information

## Figures and Tables

**Table 1:**
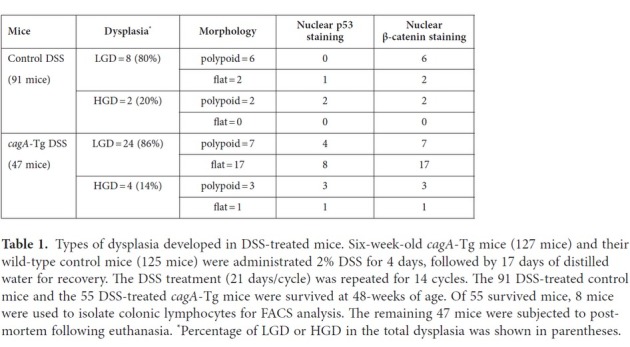
Types of dysplasia developed in DSS-treated mice.
