# Supplementary material for: Mutual reinforcement of inflammation and carcinogenesis by the Helicobacter pylori CagA oncoprotein
Source: Sci Rep. 2015 May 6;5:10024. doi: 10.1038/srep10024 (PMC4421872; doi:10.1038/srep10024)
Supplement: Supplementary Information [file srep10024-s1.pdf]

Supplementary Information

Mutual reinforcement of inflammation and carcinogenesis by the *Helicobacter pylori* CagA oncoprotein

Nobumi Suzuki, Naoko Murata-Kamiya, Kohei Yanagiya, Wataru Suda, Masahira Hattori, Hiroaki Kanda, Atsuhiro Bingo, Yumiko Fujii, Shin Maeda, Kazuhiko Koike & Masanori Hatakeyama

Supplementary Figure S1

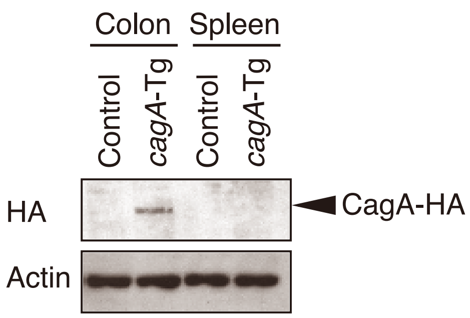

Supplementary Figure S2

a

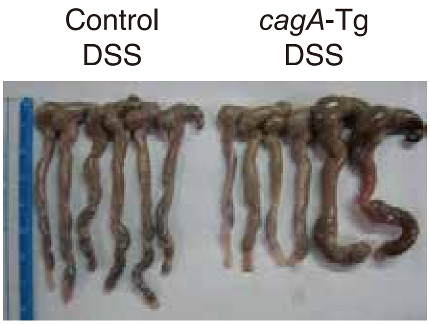

b

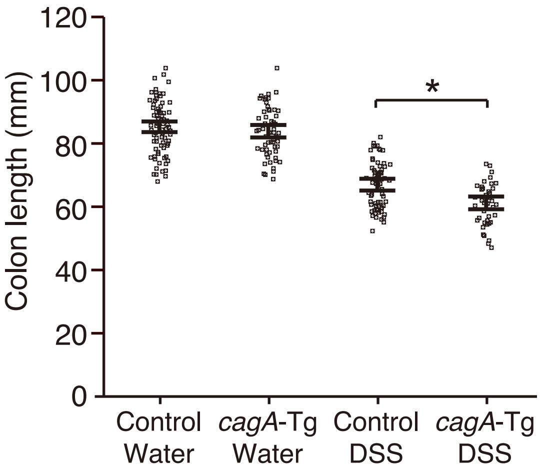

Supplementary Figure S3

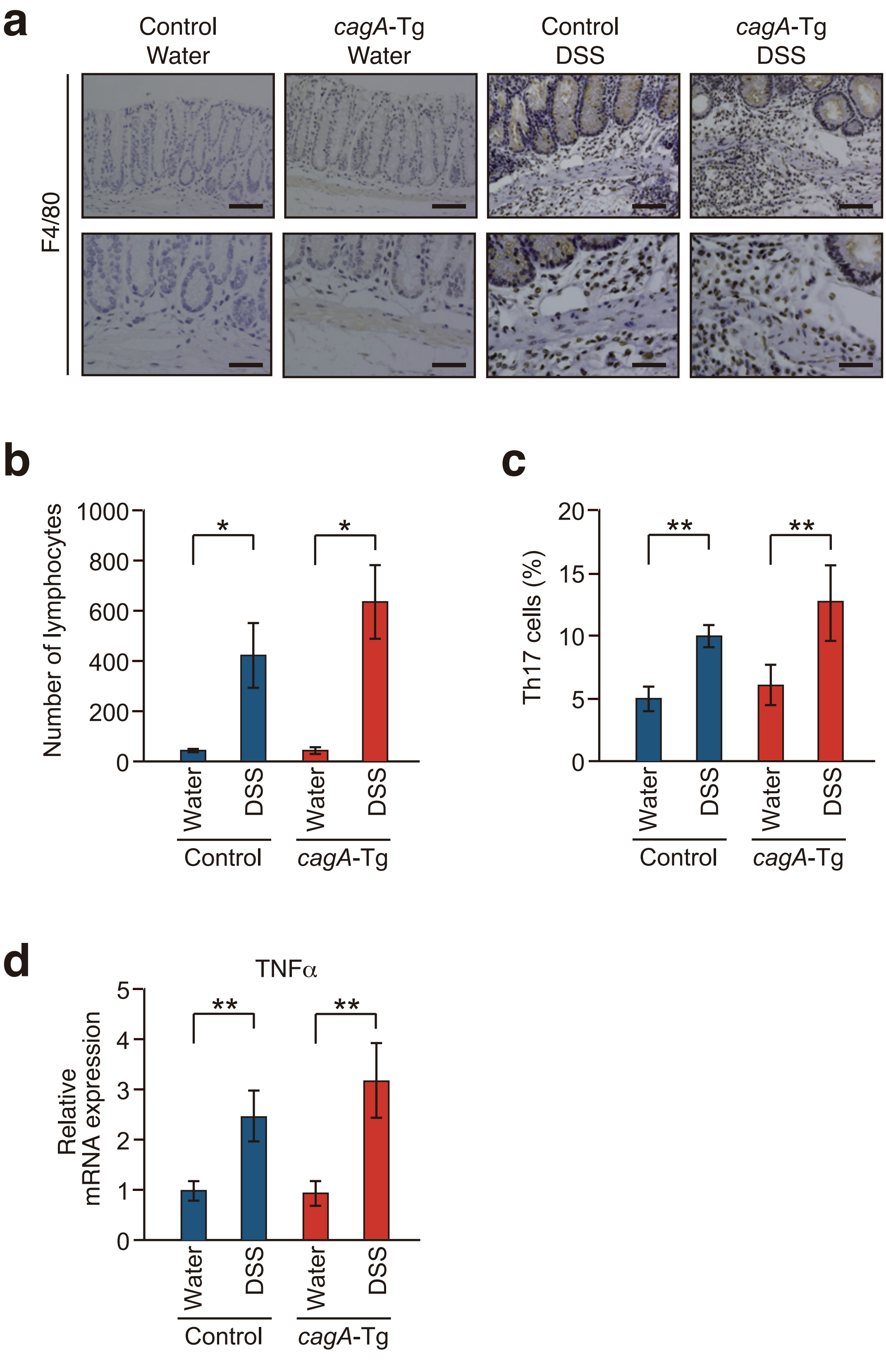

Supplementary Figure S4

a

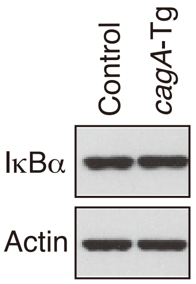

b

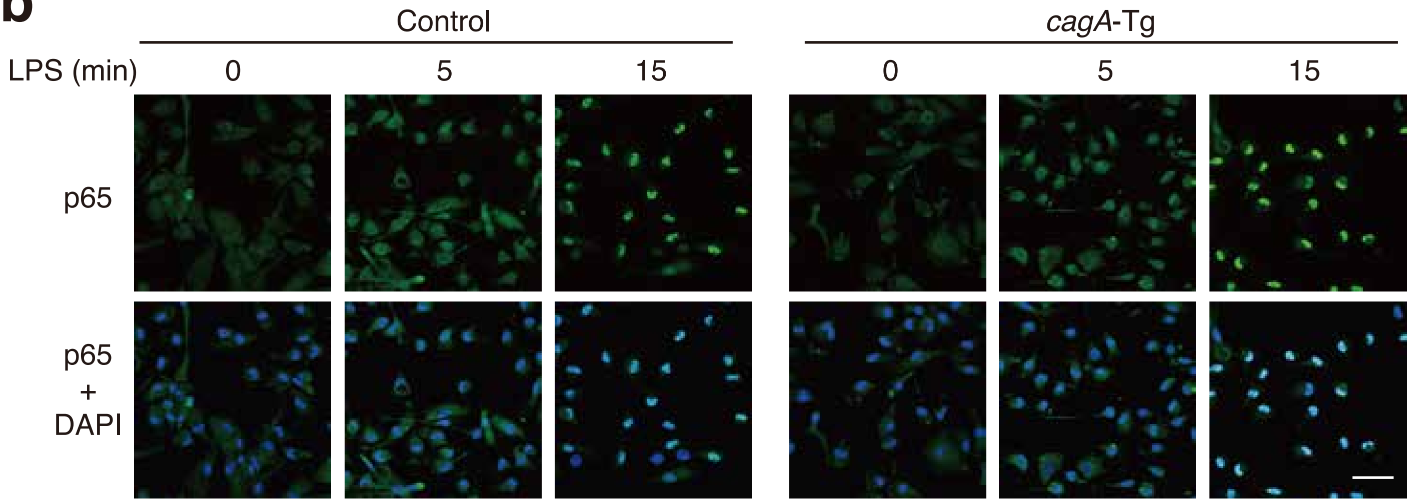

c

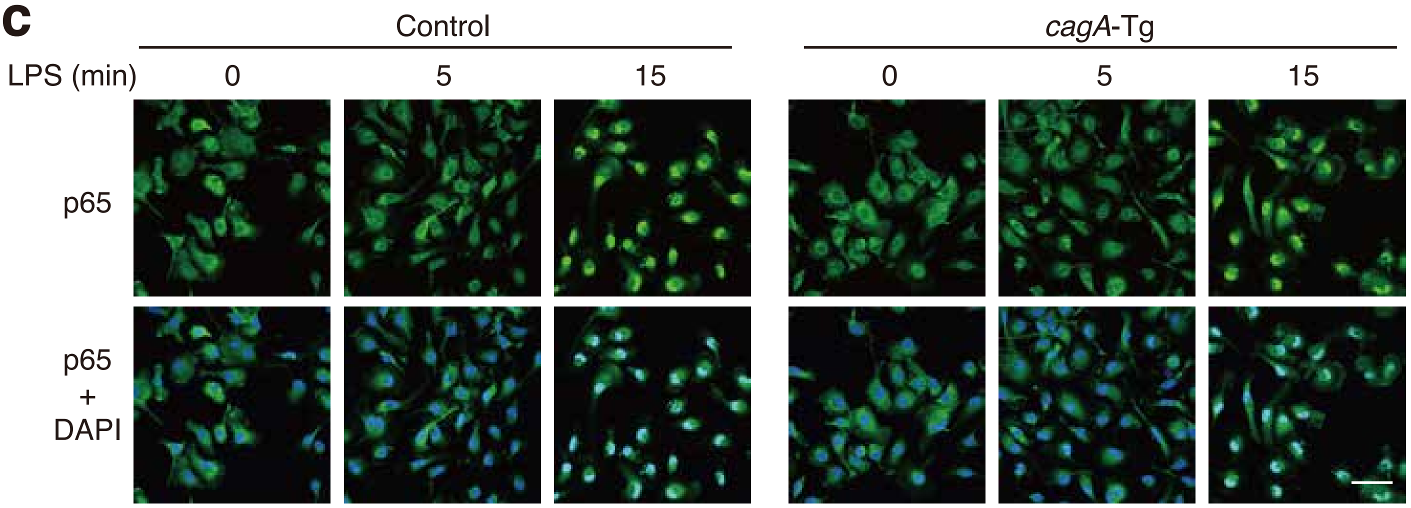

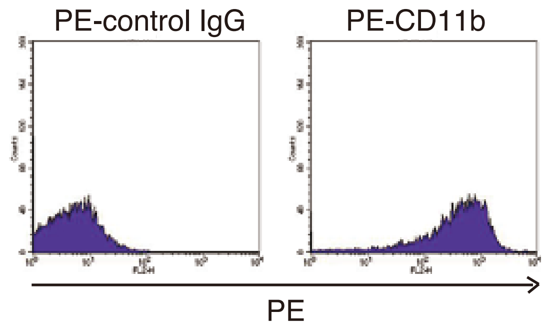

Supplementary Figure S5

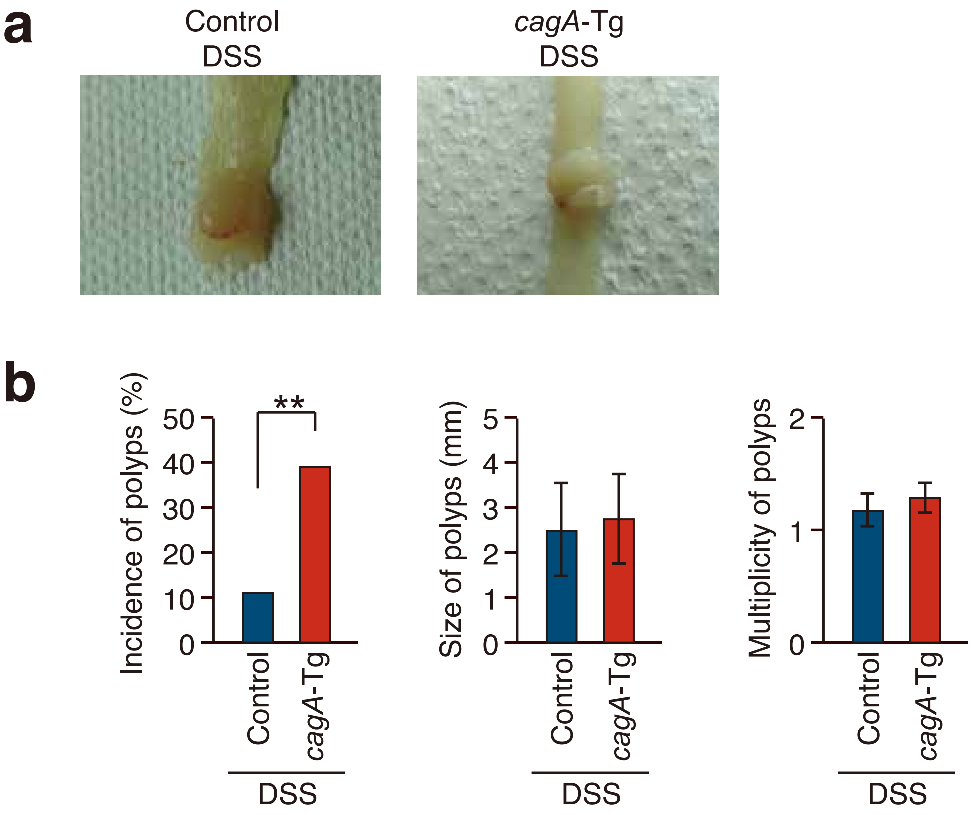

Supplementary Figure S6

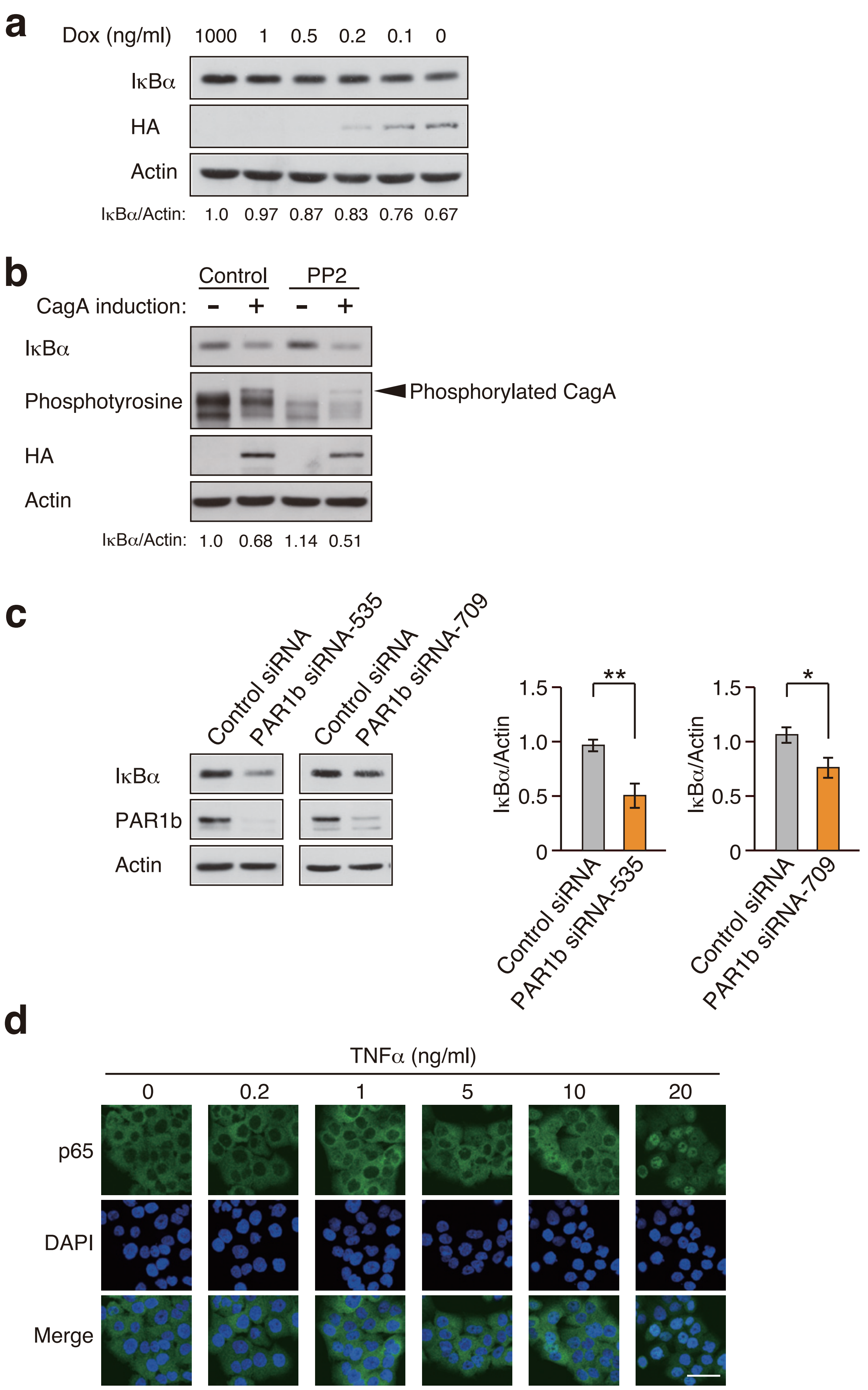

Supplementary Figure S7

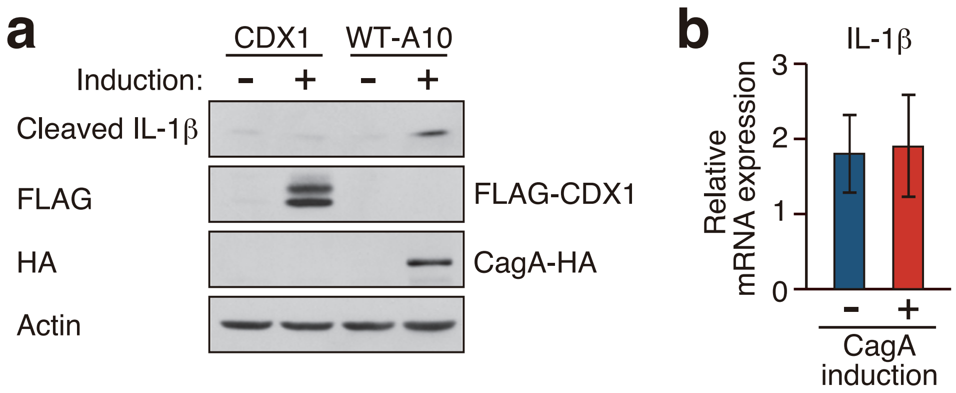

Supplementary Methods

Antibodies. Anti-IκBα (L35A5) and anti-p65 (D14E12) antibodies were from Cell signaling Technology; anti-IL-1β (ab9722) antibody was from abcam; anti-Actin (C-11) antibody was from Santa Cruz Biotechnology; anti-F4/80 antibody was from AbD Serotec; anti-HA (3F10) antibody was from Roche; anti-FLAG (M2) antibody was from SIGMA, anti-phosphotyrosine antibody (4G10) was from Upstate Biotechnology. Allophycocyanin (APC)-conjugated anti-IL17A and phycoerythrin (PE)-conjugated anti-CD11b antibodies were from BioLegend. An anti-PAR1b antibody was provided by Dr. Atsushi Suzuki (Yokohama City University, Japan).

Cell lines. WT-A10 and CDX1-inducible cells are MKN28-derived stable transfectant clones that inducibly express wild-type CagA and CDX1, respectively, using a *tet-off* system[1].

Preparation of peritoneal macrophages. Peritoneal macrophages were prepared as described previously[2]. Briefly, *cagA*-Tg mice and control mice were intraperitoneally injected with 2 ml of 4% thioglycollate bloth (DIFCO). After 3 days, mice were sacrificed and injected intraperitoneally with 5 ml of ice-cold phosphate-buffered saline (PBS). Peritoneal fluid cells were collected using a 5-ml syringe and a 21-gauge

needle. The collected cells were washed with PBS and cultured in RPMI 1640 medium supplemented with 10% fetal bovine serum (FBS). After 24 h, non-adherent cells were removed.

Preparation of bone marrow derived macrophages. Bone marrow derived macrophages (BMDM) were prepared as described previously[3]. Briefly, bone marrow cells were collected from *cagA*-Tg mice and control mice. The collected cells were cultured in RPMI 1640 medium supplemented with 10% FBS and 15% L929-cell conditioned medium that contains M-CSF for 11 days. BMDM differentiation was confirmed by flow cytometric analysis with PE-conjugated anti-CD11b antibody.

Flow cytometry. Lymphocytes isolated from mice were incubated with an allophycocyanin (APC)-conjugated anti-IL17A antibody in a fluorescence activated cell sorter (FACS) buffer (PBS containing 2% FBS, and 2 mM EDTA, 0.009% NaN_3) for 30 min. Reacted lymphocytes were analyzed using LCR Fortessa Cell Analyzer (BD Biosciences).

Quantitative RT-PCR. Total RNAs were extracted from the colon of mice or WT-A10 cells using TRIZOL Reagent (Invitrogen). cDNA generated by SuperScript II reverse transcriptase (Invitrogen) was analyzed by PCR using SYBR Premix Ex Taq (TaKaRa) and StepOnePlus

real time PCR system (Applied Biosystems). The copy numbers were normalized with values from glyceraldehyde 3-phosphate dehydrogenase (GAPDH) measured in separate real-time RT-PCR assays. The set of primers used for mouse *Gapdh* was as follows: forward, 5’-TGGTGAAGCAGGCATCTGAG-3’ and reverse, 5’-GAAGTGGAAGAGTGGGAGTTG-3’. The set of primers used for mouse *Tnfa* was as follows: forward, 5’-CATCTTCTCAAAATTCGAGTGACAA-3’ and reverse, 5’-TGGGAGTAGACAAGGTACAACCC-3’. The set of primers used for human *GAPDH* was as follows: forward, 5’-CCTCAACTACATGGTTTACATGTTCC-3’ and reverse, 5’-GAAGATGGTGATGGGATTTCCATTG-3’. The set of primers used for human *IL1B* was as follows: forward, 5’-CCACAGACCTTCCAGGAGAATG-3’ and reverse, 5’-GTGCAGTTCAGTGATCGTACAGG-3’.

RNA interference. PAR1b-specific siRNA-535 (5’-UUCACAGCUACCUCUUUCCCA-3’) and PAR1b-specific siRNA-709 (5’-GCGGAGAGGUAUUUGAUUACC-3’) were synthesized by Operon Biotechnology. Control siRNA GL2 was purchased from Cosmo Bio. WT-A10 cells were transfected with siRNA using Lipofectamine 2000 reagent (Invitrogen).

Supplementary References

1 Fujii, Y. *et al*. CDX1 confers intestinal phenotype on gastric epithelial cells via induction of stemness-associated reprogramming factors SALL4 and KLF5. *Proc. Natl. Acad. Sci. USA* **109,** 20584-20589 (2012).

2 Gallily, R. & Feldman, M. The role of macrophages in the induction of antibody in X-irradiated animals. *Immunology* **12**, 197-206 (1967).

3 Weischenfeldt, J. & Porse, B. Bone marrow-derived macrophages (BMM): isolation and applications. *Cold Spring Harb. Protoc.* **3**, Issue 12, doi: 10.1101/pdb.prot5080 (2008).

Supplementary Figure legends

Supplementary Figure S1. Immunoblot analysis of CagA expression in the colon and spleen of *cagA*-Tg mouse. Actin was used as a protein loading control.

Supplementary Figure S2. Colon length of DSS-treated mice. (a) The *cagA*-Tg mice and wild-type control mice (6 weeks of age) were administered 2% DSS in drinking water for 4 days, followed by normal drinking water for 17 days. The DSS administration was repeated for 14 cycles. Entire colon was excised from DSS-treated *cagA*-Tg mice and control mice. (b) Colon length of 48-week-old mice. Error bars, mean ± s.e.m. *P < 0.05 (ANOVA and Tukey test).

Supplementary Figure S3. Analysis of inflammatory responses in the colon of *cagA*-Tg mice. (a) Immunohistochemical staining for F4/80, a murine macrophage marker, in the colonic mucosa isolated from *cagA*-Tg mice or control mice with or without DSS treatment. Scale bars, 50 μm in upper panels, 25 μm in lower panels. (b) Number of lymphocytes per unit area in H&E staining of the colonic mucosa. Error bars, mean ± s.d. (n = 3). *P < 0.05 (Student's t-test). (c) Flow cytometric analysis of Th17 populations in the colon. Error bars, mean ± s.d. (n = 5). **P < 0.01 (Student's t-test). (d) The mRNA levels for TNFα were determined by

quantitative RT-PCR. Relative expression of *Tnfa* mRNA is shown as *Tnfa*/*Gapdh* ratio. Error bars, mean ± s.d. (n = 8). **P < 0.01 (Student's t-test).

Supplementary Figure S4. NF-κB signaling in macrophages isolated from *cagA*-Tg mice. (a) Immunoblot analysis of IκBα in peritoneal macrophages of *cagA*-Tg and control mice. Lysates prepared from the macrophages were immunoblotted with the indicated antibodies. (b) Anti NF-κB p65 immunostaining of peritoneal macrophages isolated from *cagA*-Tg mice or control mice with LPS stimulation (100 ng/ml) for the indicated times. Nuclei were visualized by DAPI. Scale bar, 40 μm. (c) Anti NF-κB p65 immunostaining of bone marrow derived macrophages (BMDM) derived from *cagA*-Tg mice or control mice with LPS stimulation (100 ng/ml) for the indicated times. Scale bar, 40 μm (upper panel). BMDM differentiation was confirmed by flow cytometric analysis with PE-conjugated anti-CD11b antibody (lower panel).

Supplementary Figure S5. Polypoid lesions developed in the colon of DSS-treated mice. (a) Six-week-old *cagA*-Tg mice (127 mice) and their wild-type control mice (125 mice) were administrated 2% DSS for 4 days, followed by 17 days of distilled water for recovery. The DSS treatment (21 days/cycle) was repeated for 14 cycles. The 91 DSS-treated control

mice and the 55 DSS-treated *cagA*-Tg mice were survived at 48-weeks of age. Of 55 survived mice, 8 mice were used to isolate colonic lymphocytes for FACS analysis. The remaining 47 mice were subjected to post-mortem following euthanasia. Macrograph of polyps developed in the colon of DSS-treated mice. (b) Effect of CagA on the development of polyps in the colon of DSS-treated mice. Incidence of polyps (left panel), size of polyps (middle panel), and multiplicity of polyps (right panel) are shown. Error bars, mean ± s.d. $^{**}P < 0.01$ (Student's t-test).

Supplementary Figure S6. Mechanistic and functional insights into CagA-mediated IκB reduction. (a) WT-A10 cells that inducibly express HA-tagged CagA by the *tet-off* system were cultured in the presence or absence of doxycycline (Dox) for 5 days. Lysates prepared were immunoblotted with the indicated antibodies. Relative amount of IκBα was calculated from the immunoblotting data. (b) WT-A10 cells were induced to express HA-tagged CagA by Dox depletion for 5 days in the presence or absence of PP2 (5 mM). Lysates prepared were immunoblotted with the indicated antibodies. Relative amount of IκBα was calculated from the immunoblotting data. (c) WT-A10 cells were transfected with control or PAR1b-specific siRNA (PAR1b-siRNA-535 or PAR1b-siRNA-709). At 72 h after transfection, cells were harvested and lysates prepared were immunoblotted with the indicated antibodies (left

panel). Relative amount of IκBα was calculated from the immunoblotting data (right panel). Error bars, mean ± s.d. ($n = 3$). *$P < 0.05$, **$P < 0.01$ (Student's t-test). (d) Anti NF-κB p65 immunostaining of WT-A10 cells treated with the indicated concentrations of TNFα for 20 min. Nuclei were visualized by DAPI. Scale bar, 40 μm.

Supplementary Figure S7. Proteasome activation by CagA in gastric epithelial cells. (a) Immunoblot analysis of mature IL-1β (cleaved IL-1β) in WT-A10 and CDX1-inducible cells cultured with or without Dox for 5 days. (b) WT-A10 cells were induced to express HA-tagged CagA by Dox depletion for 6 days. The mRNA levels for *IL1B* were determined by quantitative RT-PCR. Relative expression of *IL1B* mRNA is shown as *IL1B*/*GAPDH* ratio. Error bars, mean ± s.d. ($n = 3$).
